# Effect of the Cardio First Angel™ device on CPR indices: a randomized controlled clinical trial

**DOI:** 10.1186/s13054-016-1296-3

**Published:** 2016-05-17

**Authors:** Amir Vahedian-Azimi, Mohammadreza Hajiesmaeili, Ali Amirsavadkouhi, Hamidreza Jamaati, Morteza Izadi, Seyed J. Madani, Seyed M. R. Hashemian, Andrew C. Miller

**Affiliations:** Trauma Research Center and Nursing Faculty, Baqiyatallah University of Medical Sciences, Tehran, Iran; Loghman Clinical Research Development Center, Shahid Beheshti University of Medical Sciences, Tehran, Iran; Department of Critical Care Medicine, Mehrad Hospital, Tehran, Iran; Tobacco Prevention and Control Research Center, National Research Institute of Tuberculosis and Lung Diseases, Masih Daneshvari Hospital, Shahid Beheshti University of Medical Sciences, Tehran, Iran; Health Research Center, Baqiyatallah University of Medical Sciences, Tehran, Iran; Trauma Research Center, Baqiyatallah University of Medical Sciences, Tehran, Iran; Chronic Respiratory Diseases Research Center, National Research Institute of Tuberculosis and Lung Diseases, Masih Daneshvari Hospital, Shahid Beheshti University of Medical Sciences, Tehran, Iran; Department of Emergency Medicine, West Virginia University School of Medicine, 1 Medical Center Drive, Morgantown, WV 26506-9149 USA

**Keywords:** Cardiopulmonary resuscitation, Cardiac arrest, Cardio First Angel, CPR outcomes

## Abstract

**Background:**

A number of cardiopulmonary resuscitation (CPR) adjunct devices have been developed to improve the consistency and quality of manual chest compressions. We investigated whether a CPR feedback device would improve CPR quality and consistency, as well as patient survival.

**Methods:**

We conducted a randomized controlled study of patients undergoing CPR for cardiac arrest in the mixed medical-surgical intensive care units of four academic teaching hospitals. Patients were randomized to receive either standard manual CPR or CPR using the Cardio First Angel™ CPR feedback device. Recorded variables included guideline adherence, CPR quality, return of spontaneous circulation (ROSC) rates, and CPR-associated morbidity.

**Results:**

A total of 229 subjects were randomized; 149 were excluded; and 80 were included. Patient demographics were similar. Adherence to published CPR guidelines and CPR quality was significantly improved in the intervention group (*p* < 0.0001), as were ROSC rates (72 % vs. 35 %; *p* = 0.001). A significant decrease was observed in rib fractures (57 % vs. 85 %; *p* = 0.02), but not sternum fractures (5 % vs. 17 %; *p* = 0.15).

**Conclusions:**

Use of the Cardio First Angel™ CPR feedback device improved adherence to published CPR guidelines and CPR quality, and it was associated with increased rates of ROSC. A decrease in rib but not sternum fractures was observed with device use. Further independent prospective validation is warranted to determine if these results are reproducible in other acute care settings.

**Trial registration:**

ClinicalTrials.gov identifier: NCT02394977. Registered on 5 Mar 2015.

## Background

Effective chest compression remains the cornerstone of successful cardiopulmonary resuscitation (CPR) and is vital for patient survival and good neurological recovery [1–12]. International guidelines note the critical importance of the quality of manual chest compression components, including hand position, position of rescuer and victim, and compression rate and depth [9, 13–20]. Several devices have been developed to improve the consistency and quality of chest compressions [8, 14, 18, 21]. These include active compression-passive decompression (ACPD) devices and active compression-active decompression devices. Some of these devices are coupled to feedback technology to help guide or inform the rescuer about CPR. CPR feedback technology ranges in complexity from a simple metronome to more complex devices that monitor and provide real-time audiovisual feedback [22–24]. Some devices are designed for use by both medical personnel and laypersons [25–29]. While guidelines currently do not recommend any of these circulatory adjuncts, owing to insufficient data, some are being used routinely in resuscitation as alternatives to standard manual chest compressions [8, 30, 31].

The feedback devices may be divided into those associated or not with automated external defibrillators (AEDs). The non-AED ACPD devices are small, lightweight, portable devices that are positioned between the patient and rescuer. These devices are positioned on the patient’s chest where the rescuer’s hands would normally be positioned during manual CPR, and compressions are performed on the device. To date, four non-AED-associated and five AED-associated CPR feedback devices have been tested in clinical and training environments [14].

Feedback devices rely on either pressure or acceleration measurements as surrogates for compression depth to determine compression adequacy. The Cardio First Angel™ (CFA; INOTECH, Nubberg, Germany) is an ACPD CPR feedback system designed for use by both laypersons and healthcare professionals. We sought to determine if the addition of CFA to routine manual CPR would impact the quality and consistency of CPR and patient survival.

## Methods

### Study design and settings

We conducted a randomized, controlled, single-blind study of patients undergoing CPR for cardiac arrest in the mixed medical-surgical intensive care units (ICUs) of four academic teaching hospitals in Tehran, Iran, between 1 June and 31 October 2014. Patients were randomized in the emergency department at the time of ICU admission. The intervention and control groups were labeled groups 1 and 2, respectively. The assessment tool was scored by the principal investigator and an ICU nurse. The data analyzer was blinded to group randomization and was not present during CPR.

### Patient population

Patients were eligible for study participation if they met the following criteria: (1) age ≥18 years, (2) admitted to the ICU, (3) full code status, and (4) informed consent was obtained from the patient, legal guardian, or healthcare surrogate upon ICU admission (before cardiac arrest event). Patients with any limitation of code status including but not limited to “no code” or “do not resuscitate” and “do not intubate” were excluded from study participation. Decisions to cease resuscitation efforts were made in accordance with American Heart Association guidelines and included (1) >30 minutes of resuscitation process without any events of ventricular fibrillation or ventricular tachycardia, (2) asystole due to irreversible cause, (3) initial rhythm of asystole, (4) injury not compatible with life, or (5) severity of comorbidities, in the presence of normothermia.

**Fig. 1:**
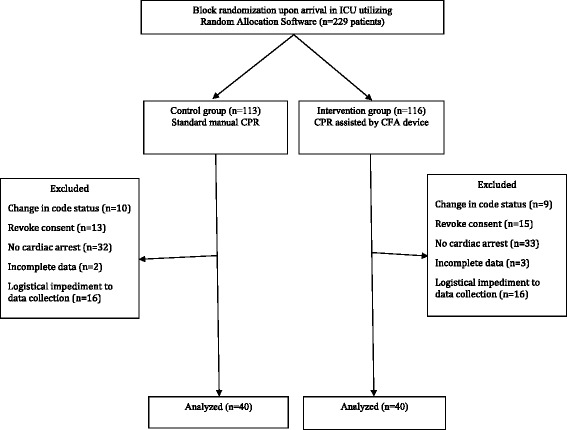
Flowchart of patient enrollment. *CFA* Cardio First Angel™, *CPR* cardiopulmonary resuscitation, *ICU* intensive care unit

Patients were enrolled consecutively from among available admitted ICU patients. Randomization was accomplished using Random Allocation Software© (Informer Technologies, Inc., Madrid, Spain) (Fig. 1). Block randomization was performed with a computer-generated random number list by an expert statistician who had no clinical involvement in the trial. Allocation assignment was accomplished through confidential communication between the patient’s nurse and a third party who was not involved in the recruitment process.

### Ethical considerations

All parts of the study were reviewed according to the Consolidated Standards of Reporting Trials (CONSORT) statement (Fig. 1) [32, 33]. The trial is registered with ClinicalTrials.gov with the identifier NCT02394977. The protocol was approved by the institutional review boards at the four participating medical centers in Tehran: Baqiyatallah Hospital, Masih Daneshvari Hospital, Loghman Hakim Hospital, and Shahid Modares Hospital. Consent covered both study participation and consent to publish the findings. Informed consent was required. Surrogate consent from the patient’s legal guardian or healthcare proxy was permitted in cases where the patient did not have decision-making capacity.

### Intervention

Chest compressions were performed by ICU nurses. Before the start of the study, all ICU nurses at approved study sites received standardized CPR training in accordance with published guidelines, in addition to formal training with the CFA device [9, 20]. Upon CPR initiation, patients in the control group received CPR in accordance with published guidelines, and patients in the intervention group received CPR in accordance with published guidelines using the CFA device per the manufacturer’s instructions. Compressor rotation was not standardized and was dependent on clinical staffing as well as the physical health, condition, and stamina of those providing compressions. Invasive hemodynamics were not measured during the resuscitation, as this was outside the scope of this study.

### Cardio First Angel ™ device

**Fig. 2:**
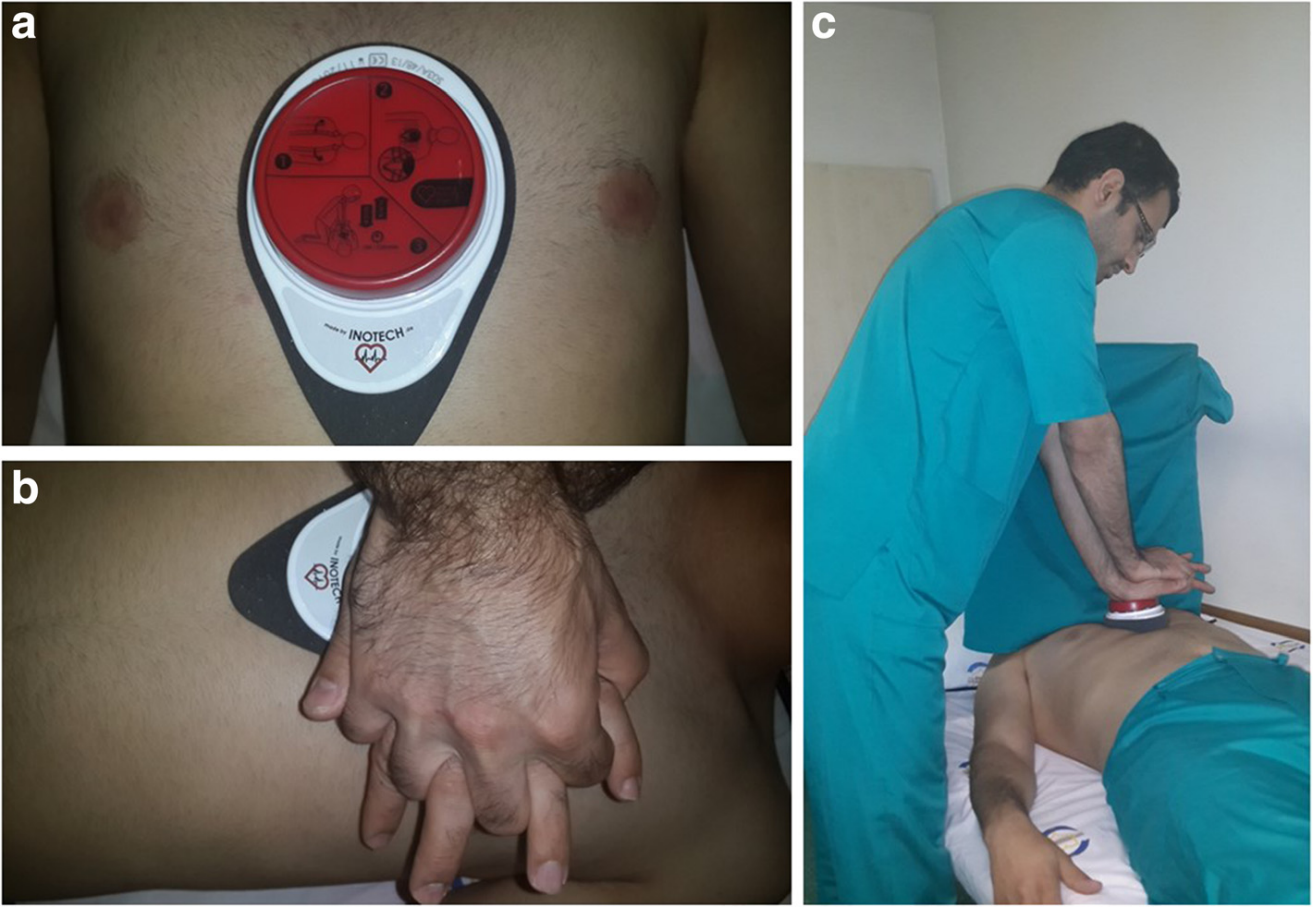
Illustration of proper positioning of the Cardio First Angel™ device. **a** The device is positioned over the lower one-third of the sternum. The rescuer-side portion consists of a red, round push-button that displays a pictogram illustrating proper device use. **b** The push-button fits in the palm of the rescuer’s hand as it would lie on the sternum during manual cardiopulmonary resuscitation (CPR). **c** The rescuer should maintain straight arms and back and flex at the waist as in standard manual CPR

The Cardio First Angel™ is a lightweight device (130 g, 4.5 oz) that consists of three components (Fig. 2). The rescuer-side portion consists of a red, round push-button that fits in the palm of the rescuer’s hand as it would lie on the sternum during manual CPR. This rescuer-side component displays a pictogram illustrating proper device use. The center unit is composed of a stable plastic base that contains a complex arrangement of springs to transfer force to the patient’s thorax. The patient side of the device consists of liquid-absorbent polyurethane foam that disperses the compression force evenly across the device footprint. Application of 400 ± 30 N of force (41 kg or 90 lb of pressure), which correlates to a sternum compression depth of 50–60 mm, is followed by an audible “click” sound to alert the rescuer to cease compression. The “click” sound is also audible upon spring decompression, alerting the rescuer to resume compression.

### Data collection

The data collection tool included both demographic and CPR-specific variables. The assessment tools were developed during two 90-minute meetings by a consensus multidisciplinary panel consisting of 11 physicians representing critical care (*n* = 3), anesthesia (*n* = 3), cardiology (*n* = 2), pulmonology (*n* = 1), internal medicine (*n* = 1), and forensic medicine (*n* = 1), in addition to 10 critical care nurses. The assessment tool was scored by the principal investigator and an ICU nurse. Tool validation was performed using the Delphi technique. Assessment of interrater reliability yielded a κ score of 0.9.

Collected demographic data included age, sex, invasive mechanical ventilation status upon code onset (yes or no), ICU length of stay, diagnoses, presence of known osteoporosis (yes or no), and incidence of sternum or rib fractures (on x-ray or autopsy). Also recorded were initial rhythm and therapeutic agents administered during the resuscitation. Time of resuscitation occurrence (morning, midday, evening, or night) and nurse’s level (years) of critical care nursing experience were recorded. CPR effectiveness was evaluated on a scale of 0 (lowest) to 10 (highest) based upon patient position, CPR event frequency, presence of a working intravenous line, use of a CPR board or deflation of air mattress, environmental management, CPR duration, and return of spontaneous circulation (ROSC). Observation of the adherence to CPR guidelines on a scale of 0 (lowest) to 10 (highest) was recorded using a checklist. Assessment of guideline adherence was based upon timeliness of compression initiation, effective team coordination and observation of preassigned roles, compression rate, compression depth, rescuer position, airway management, medication administration, and appropriate use of defibrillation and pacing. Both checklists were validated on the basis of content validity ratio (0.54) and content validity index (0.89).

### Data analysis

All analyses were performed using IBM SPSS 22.0 software (IBM, Armonk, NY, USA). Frequency (percent) and mean (SD) were presented for qualitative and quantitative variables. The normality of study variables was assessed with the one-sample Kolmogorov-Smirnov test. The median value (quartile 1 to quartile 3) was presented as a summary statistic for nonnormative variables, including CPR duration, nurse satisfaction with CPR quality, CPR evaluation, and observation of CPR guidelines. Frequency was presented as a summary statistic for ROSC and incidence of rib and sternum fractures. Non-Gaussian variables were compared with the Mann-Whitney *U* test, *χ*^2^ test, or Fisher’s exact test as appropriate. Demographic variables were compared using a *t* test, *χ*^2^ test, or Fisher’s exact test as appropriate. Statistical significance was defined as a *p* value <0.05.

## Results

Of 229 consecutive patients eligible for study enrollment, 149 were excluded and 80 were included (Fig. 1). Patient demographics were similar between groups (Table 1). The percentage of female patients was similar between groups (55 % vs. 65.5 %; *p* = 0.56) as was patient age (59 ± 13 years vs. 62 ± 13 years; *p* = 0.32). Admission diagnoses were similar (Table 2). All cases of cardiac arrest occurred in the ICU. Identified initial rhythms compared between intervention and control groups included asystole (30 % vs. 33 %), ventricular tachycardia (45 % vs. 45 %), ventricular fibrillation (12.5 % vs. 12.5 %), and bradyarrhythmia (12.5 % vs. 10 %).

**Table 1:** Demographic variables

Variable	Total (*N* = 80)		Intervention (*N* = 40)		Control (*N* = 40)		
	Mean ± SD	*n* (%)	Mean ± SD	*n* (%)	Mean ± SD	*n* (%)	*p* Value
Age, years	61.10 ± 13.31		59.62 ± 13.27		62.57 ± 13.35		0.32^a^
ICU length of stay, days	22.45 ± 15.41		18.22 ± 15.42		26.68 ± 14.37		0.28^a^
Nurse ICU experience, years	19.57 ± 5.51		19.3 ± 5.45		19.85 ± 5.63		0.65^a^
Sex, female		49 (61.3)		22 (55.0)		27 (65.5)	0.6^b^
Intubated before CPR event		46 (57.5)		21 (52.5)		25 (62.5)	0.49^b^
Multiorgan dysfunction, yes		44 (55.0)		22 (55.0)		22 (55.0)	1.00^b^
Osteoporosis, no		69 (86.3)		36 (90.0)		33 (82.0)	0.52^c^

*CPR* cardiopulmonary resuscitation, *ICU* intensive care unit

^a^ *t* test

^b^χ^2^ test with Yates correction

^c^Fisher’s exact test

**Table 2:** Distribution of admission diagnoses

Diagnosis category	CFA, *n* (%)	Controls, *n* (%)	*p* Value
Trauma	6 (15)	5 (12.5)	0.75
Neurological	10 (25)	9 (22.5)	0.79
Renal	3 (7.5)	3 (7.5)	1.00
Cancer	9 (22.5)	10 (25)	0.79
Respiratory	6 (15)	5 (12.5)	0.75
Abdominal infection	6 (15)	8 (20)	0.56

Use of resuscitation therapies was similar between groups (Table 3). No significant difference was observed between groups in total dose of electricity administered for defibrillation. No significant difference in either the rate of administration or the dose of administered epinephrine, vasopressin, atropine, amiodarone, calcium gluconate, or sodium bicarbonate was observed (Table 3). Although the frequency of lidocaine administration was similar between groups, there was a significant increase in dose administered in the intervention group (*p* < 0.0001).

**Table 3:** Comparison of resuscitation treatments^a^

	Treatment administered			Treatment dose, control		
Agent	CFA, *n* (%)	Control *n* (%)	*p* Value^b^	CFA, Median (Mean)	Median (Mean)	*p* Value^c^
Electricity, J	31 (78)	28 (70)	0.61	600 (600)	600 (607)	0.77
Epinephrine, mg	40 (100)	40 (100)	NS	4 (4)	4 (4)	NS
Vasopressin, U	21 (53)	18 (45)	0.65	40 (40)	40 (40)	NS
Atropine, mg	10 (25)	11 (28)	1.00	1 (0.9)	1 (0.7)	0.14
Lidocaine, mg	13 (33)	14 (35)	1.00	200 (190)	120 (124)	<0.0001
Amiodarone, mg	16 (40)	15 (38)	1.00	450 (384)	450 (390)	0.85
Sodium bicarbonate, mEq	8 (20)	15 (38)	0.14	112 (106)	89 (94)	0.48
Calcium gluconate, g	3 (7.5)	4 (10)	1.00	1 (1)	1 (1)	NS
Magnesium sulfate, g	6 (15)	3 (8)	0.48	2 (2)	2 (1)	NS

*CFA* Cardio First Angel™, *NS* not significant

^a^Numbers rounded to nearest whole number

^b^Fisher’s exact test

^c^Wilcoxon test

When we considered risk factors for CPR-associated morbidity, we found that the percentage of patients with osteoporosis was similar between groups (90 % vs. 82 %; *p* = 0.52). Moreover, the shift of CPR occurrence (morning, midday, evening, night) was similar between groups (*p* = 0.78).

Adherence to published CPR guidelines as well as CPR quality were improved in the intervention group (*p* < 0.0001) (Table 4). No significant difference in CPR duration was observed between groups (34 minutes vs. 36 minutes; *p* = 0.076) (Table 5). ROSC was observed more frequently in the intervention group (72 % vs. 35 %; *p* = 0.001). A significant decrease in rib fractures was identified between groups (57 % vs. 85 %; *p* = 0.02). No significant difference in sternum fractures was observed (5 % vs. 17 %; *p* = 0.15).

**Table 4:** Summary statistics and the results of the tests for comparing the groups for three study variables

Variable	Total (*n* = 80)		Intervention (*n* = 40)		Control (*n* = 40)		
	Median	(Q1–Q3)	Median	(Q1–Q3)	Median	(Q1–Q3)	*p* Value^a^
CPR duration	34	(32–43)	34	(32–36)	36	(33–44)	0.076
CPR evaluation score	7	(6–9)	9	(9–9.75)	6	(5–6)	<0.0001
CPR guideline observation score	7.5	(5–9)	9	(8–10)	5	(5–6)	<0.0001

*CPR* cardiopulmonary resuscitation

^a^Mann-Whitney *U* test

**Table 5:** Summary of CPR event survival and associated morbidity

Variable	Intervention (*n* = 40)	Control (*n* = 40)	
	Frequency (%)	Frequency (%)	*p* Value^a^
Return of spontaneous circulation	29 (72.5)	14 (35)	0.001
Sternum fracture	2 (5)	7 (17.5)	0.15
Any rib fracture	23 (57.5)	33 (82.5)	0.02
Number of rib fractures			
0	17 (42.5)	7 (17.5)	
1	16 (40)	6 (15)	
2	6 (15)	15 (37.5)	
3	1 (2.5)	9 (22.5)	
4	0	2 (5)	
5	0	1 (2.5)	

*CPR* cardiopulmonary resuscitation

^a^χ^2^ test

## Discussion

Effective chest compression remains the cornerstone of successful CPR and is vital for survival and good neurological recovery [1–12]. As such, the quality of chest compression remains a focal point of international guidelines [9, 13–20]. Both compression rate and depth are of critical importance, with compression frequencies of <75 and >125 per minute being associated with decreased incidence of ROSC [11]. Moreover, use of proper compression force and depth is important to minimize CPR-associated injuries [8]. A number of CPR adjunct devices have been developed to improve the consistency and quality of chest compressions [8, 14, 18, 21]. Many of these devices incorporate an auditory tool to guide the compression rate. This is based on a number of clinical studies in which researchers noted that audible rate guidance during chest compressions may improve CPR performance [34, 35]. With few exceptions [36], researchers in early simulation studies of CPR feedback devices have generally reported improved CPR quality as defined by compression rate and depth [25, 37–40]. To date, only the AED-associated CPR feedback devices have been tested clinically, and reports on their efficacy for improving CPR quality have been conflicting [36, 40, 41]. Even when chest compression frequency does not differ significantly from the ideal set out in the guidelines, analysis of “no-flow times” may be significantly longer for the first CPR cycle [42]. Moreover, devices based on accelerometer technology are not accurate on surfaces that are not firm and flat. They also do not correct for the compression of an underlying mattress, leading to significant undercompression of the chest during CPR [24, 43]. Both the increase in “no-flow times” and undercompression may be clinically relevant impediments to high-quality CPR [44, 45].

Before the present study, four non-AED CPR feedback devices had been tested in clinical or medical simulation environments. Although none have been tested clinically, each has been tested in simulated resuscitation environments. The CPRmeter™ (Laerdal, Wappingers Falls, NY, USA) uses an accelerometer to calculate compression depth, as well as a pressure sensor to determine the adequacy of chest wall release or recoil during decompression. Although early reports suggested that the CPRmeter™ significantly improved chest compression quality performed by students inexperienced in CPR [25], a later report suggested that compression quality was inferior to standard basic life support. Moreover, device use caused a substantial delay in CPR initiation [26]. Similar findings were reported with use of the Zoll Pocket CPR™ device (Zoll Medical Corp., Chelmsford, MA, USA) [26]. Although statistically significant, it is unclear if this delay in CPR initiation is clinically significant.

Both the CPREzy™ (Health Affairs Ltd., Berkhamsted, UK) and CPR PLUS™ devices (Kelly Medical Products, Princeton, NJ, USA) rely on direct measurements of pressure. The CPREzy™ device is battery-powered and relies on light-emitting diodes to notify the rescuer when appropriate compression force has been applied. In one study, the CPREzy™ was associated with a higher number of incorrectly placed compressions (26 % vs. 3.9 % standard CPR; *p* < 0.001) [46]. This was largely due to a higher number of low compressions (26 % of total compressions for CPREzy vs. 1 % for standard CPR; *p* < 0.001) [46]. Use of the CPREzy™ device may be associated with rescuer fatigue, as it is associated with a 21–26.5 % increase in rescuer work [47]. Moreover, use was associated with a significant risk of soft tissue injury of the rescuer’s hand [46].

The CPR PLUS™ device measures compressive pressure and reports this measure to the rescuer via a meter with a movable needle. In a medical simulation study, use of the CPR PLUS™ device resulted in an improved number of correctly applied compressions and reduction in excessive pressure application compared with standard CPR [39]. Limitations of this device include a meter that is difficult to read in real time and a shape that does not fit the rescuer’s hand in an ergonomic fashion.

The Cardio First Angel™ is a non-AED CPR feedback device that uses direct pressure measurement to provide an auditory stimulus to guide both compression and decompression. When it is used properly, rescuers may deliver approximately 100–120 compressions per minute. By measuring pressure directly rather than relying on a calculated pressure based upon movement of an accelerometer, it avoids the limitations of the Laerdal CPRmeter™ and Zoll Pocket CPR™ devices. The Cardio First Angel™ device improves on the CPREzy™ and CPR PLUS™ devices by employing an auditory stimulus to direct compression-decompression, which frees the rescuer to look in other directions and potentially participate in other aspects of the resuscitation. This may be advantageous for environments with few (one to three) rescuers as compared with acute care environments where considerably more assistance may be available.

To our knowledge, this is the first study to show improved ROSC with the use of a non-AED CPR feedback device. Multiple reasons likely exist for the larger-than-expected increase in mortality, and it remains unclear how much of the increase is directly attributable to use of the device. Interestingly, adherence to CPR guidelines was significantly higher in the intervention group and likely also contributed in part to the improved mortality. It remains unclear whether device use directly promoted improved adherence to CPR guidelines or whether an unmeasured variable such as hypervigilance on the part of providers using a new CPR device contributed. Regardless of these considerations, the findings of the present study warrant prospective independent validation, ideally with direct comparison to other devices and standard CPR.

### Limitations

CPR guideline adherence was significantly higher in the intervention group. It remains unclear to what degree this is a direct reflection of device use vs. other unmeasured variables. Additionally, although rates of lidocaine administration were similar between groups, the dose was significantly higher in the intervention group. Although this relates to a relatively small percentage of the study patients, both the reason for and the impact of this discrepancy remains unclear.

One criticism of devices that encourage right-not right, one-size-fits-all CPR is that they do not account for complex changes that occur during CPR. Like other CPR feedback devices, the CFA accounts for neither complex changes in chest wall compliance and elasticity nor the compressibility of the surface on which the patient is lying (e.g., a mattress). Additionally, there are unique aspects to the ICUs at the enrolling sites in the present study that limit the generalizability of the findings. For example, only experienced ICU nurses were observed. It remains unclear to what degree the level of nursing experience impacted findings or whether these findings are generalizable to less experienced nurses or nurses in other acute care environments.

Last, the incidence of cardiac arrest was higher than one might anticipate at academic medical centers in Europe or the United States. The reason for this is likely multifactorial. It may in part relate to the availability and allocation of ICU resources. For example, Iran has far fewer ICU beds per 100,000 population than the United States does [48–50]. Additionally, demand may dictate that patients with “do not resuscitate” status or those with more minor critical illnesses may be managed outside the ICU. This may potentially result in a more acutely ill ICU population at higher risk for cardiac arrest.

## Conclusions

Use of the Cardio First Angel™ CPR feedback device improved adherence to published CPR guidelines, CPR quality, and nurse satisfaction and was associated with increased rates of ROSC in patients with cardiac arrest in four academic ICUs. Although no difference in sternum fractures was observed, a decrease in rib fractures was observed with device use. Further independent prospective validation is warranted to determine if our results are reproducible in other acute care settings.

## Abbreviations

ACPD: active compression-passive decompression
AED: automated external defibrillator
CFA: Cardio First Angel™
CONSORT: Consolidated Standards of Reporting Trials
CPR: cardiopulmonary resuscitation
ICU: intensive care unit
NS: not significant
ROSC: return of spontaneous circulation

## References

[1] Valenzuela TD, Kern KB, Clark LL, Berg RA, Berg MD, Berg DD (2005). Interruptions of chest compressions during emergency medical systems resuscitation. Circulation.

[2] Allan KS, Wong N, Aves T, Dorian P (2013). The benefits of a simplified method for CPR training of medical professionals: a randomized controlled study. Resuscitation.

[3] Ewy GA (2007). Cardiac arrest—guideline changes urgently needed. Lancet.

[4] Roosa JR, Vadeboncoeur TF, Dommer PB, Panchal AR, Venuti M, Smith G (2013). CPR variability during ground ambulance transport of patients in cardiac arrest. Resuscitation.

[5] Glatz AC, Nishisaki A, Niles DE, Hanna BD, Eilevstjonn J, Diaz LK (2013). Sternal wall pressure comparable to leaning during CPR impacts intrathoracic pressure and haemodynamics in anaesthetized children during cardiac catheterization. Resuscitation.

[6] Christenson J, Andrusiek D, Everson-Stewart S, Kudenchuk P, Hostler D, Powell J (2009). Chest compression fraction determines survival in patients with out-of-hospital ventricular fibrillation. Circulation.

[7] Idris AH, Guffey D, Aufderheide TP, Brown S, Morrison LJ, Nichols P (2012). Relationship between chest compression rates and outcomes from cardiac arrest. Circulation.

[8] Miller AC, Rosati SF, Suffredini AF, Schrump DS (2014). A systematic review and pooled analysis of CPR-associated cardiovascular and thoracic injuries. Resuscitation.

[9] Sayre MR, Koster RW, Botha M, Cave DM, Cudnik MT, Handley AJ (2010). Part 5: Adult basic life support. 2010 International Consensus on Cardiopulmonary Resuscitation and Emergency Cardiovascular Care Science with Treatment Recommendations [published correction appears in Circulation. 2013;128(19):e393]. Circulation.

[10] Nolan JP, Perkins GD, Soar J (2012). Chest compression rate: where is the sweet spot?. Circulation.

[11] Kovacs A, Vadeboncoeur TF, Stolz U, Spaite DW, Irisawa T, Silver A, Bobrow BJ (2015). Chest compression release velocity: Association with survival and favorable neurologic outcome after out-of-hospital cardiac arrest. Resuscitation..

[12] Smekal D, Lindgren E, Sandler H, Johansson J, Rubertsson S (2014). CPR-related injuries after manual or mechanical chest compressions with the LUCAS™ device: a multicentre study of victims after unsuccessful resuscitation. Resuscitation.

[13] Ayala U, Eftestøl T, Alonso E, Irusta U, Aramendi E, Wali S (2014). Automatic detection of chest compressions for the assessment of CPR-quality parameters. Resuscitation.

[14] Gruber J, Stumpf D, Zapletal B, Neuhold S, Fischer H (2012). Real-time feedback systems in CPR. Trends Anaesth Crit Care.

[15] Stiell IG, Brown SP, Christenson J, Cheskes S, Nichol G, Powell J (2012). What is the role of chest compression depth during out-of-hospital cardiac arrest resuscitation?. Crit Care Med.

[16] Cheskes S, Schmicker RH, Christenson J, Salcido DD, Rea T, Powell J (2011). Perishock pause: an independent predictor of survival from out-of-hospital shockable cardiac arrest. Circulation.

[17] Fried DA, Leary M, Smith DA, Sutton RM, Niles D, Herzberg DL (2011). The prevalence of chest compression leaning during in-hospital cardiopulmonary resuscitation. Resuscitation.

[18] Abella BS, Sandbo N, Vassilatos P, Alvarado JP, O’Hearn N, Wigder HN (2005). Chest compression rates during cardiopulmonary resuscitation are suboptimal: a prospective study during in-hospital cardiac arrest. Circulation.

[19] Mottram AR, Page RL (2012). Advances in resuscitation. Circulation.

[20] Koster RW, Baubin MA, Bossaert LL, Caballero A, Cassan P, Castrén M (2010). European Resuscitation Council Guidelines for Resuscitation 2010 Section 2. Adult basic life support and use of automated external defibrillators. Resuscitation.

[21] Sugerman NT, Edelson DP, Leary M, Weidman EK, Herzberg DL, Vanden Hoek TL (2009). Rescuer fatigue during actual in-hospital cardiopulmonary resuscitation with audiovisual feedback: a prospective multicenter study. Resuscitation.

[22] Fischer H, Gruber J, Neuhold S, Frantal S, Hochbrugger E, Herkner H (2011). Effects and limitations of an AED with audiovisual feedback for cardiopulmonary resuscitation: a randomized manikin study. Resuscitation.

[23] Fischer H, Neuhold S, Zapletal B, Hochbrugger E, Koinig H, Steinlechner B (2011). A manually powered mechanical resuscitation device used by a single rescuer: a randomised controlled manikin study. Resuscitation.

[24] Segal N, Laurent F, Maman L, Plaisance P, Augustin P (2012). Accuracy of a feedback device for cardiopulmonary resuscitation on a dental chair. Emerg Med J.

[25] Buleon C, Parienti JJ, Halbout L, Arrot X, De Facq RH, Chelarescu D (2013). Improvement in chest compression quality using a feedback device (CPRmeter): a simulation randomized crossover study. Am J Emerg Med.

[26] Zapletal B, Greif R, Stumpf D, Nierscher FJ, Frantal S, Haugk M (2014). Comparing three CPR feedback devices and standard BLS in a single rescuer scenario: a randomised simulation study. Resuscitation.

[27] Yeung J, Meeks R, Edelson D, Gao F, Soar J, Perkins GD (2009). The use of CPR feedback/prompt devices during training and CPR performance: a systematic review. Resuscitation.

[28] Skorning M, Beckers SK, Brokmann JC, Rörtgen D, Bergrath S, Veiser T (2010). New visual feedback device improves performance of chest compressions by professionals in simulated cardiac arrest. Resuscitation.

[29] Papalexopoulou K, Chalkias A, Dontas I, Pliatsika P, Giannakakos C, Papapanagiotou P (2014). Education and age affect skill acquisition and retention in lay rescuers after a European Resuscitation Council CPR/AED course. Heart Lung.

[30] Shuster M, Lim SH, Deakin CD, Kleinman ME, Koster RW, Morrison LJ (2010). Part 7: CPR techniques and devices: 2010 International Consensus on Cardiopulmonary Resuscitation and Emergency Cardiovascular Care Science with Treatment Recommendations. Circulation.

[31] Hazinski MF, Nolan JP, Billi JE, Böttiger BW, Bossaert L, de Caen AR (2010). Part 1: Executive summary: 2010 International Consensus on Cardiopulmonary Resuscitation and Emergency Cardiovascular Care Science with Treatment Recommendations. Circulation.

[32] Hopewell S, Altman DG, Moher D, Schulz KF (2008). Endorsement of the CONSORT Statement by high impact factor medical journals: a survey of journal editors and journal ‘Instructions to Authors’. Trials.

[33] Moher D, Schulz KF, Altman DG (2001). The CONSORT statement: revised recommendations for improving the quality of reports of parallel group randomized trials. BMC Med Res Methodol.

[34] Berg R, Sanders A, Milander M, Tellez D, Liu P, Beyda D (1993). Efficacy of audio-prompted rate guidance in improving resuscitator performance of cardiopulmonary resuscitation on children. Acad Emerg Med.

[35] Kern KB, Sanders AB, Raife J, Milander MM, Otto CW, Ewy GA (1992). A study of chest compression rates during cardiopulmonary resuscitation in humans: the importance of rate-directed chest compressions. Arch Intern Med.

[36] Lyngeraa TS, Hjortrup PB, Wulff NB, Aagaard T, Lippert A (2012). Effect of feedback on delaying deterioration in quality of compressions during 2 minutes of continuous chest compressions: a randomized manikin study investigating performance with and without feedback. Scand J Trauma Resusc Emerg Med.

[37] Noordergraaf GJ, Drinkwaard BW, van Berkom PF, van Hemert HP, Venema A, Scheffer GJ (2006). The quality of chest compressions by trained personnel: the effect of feedback, via the CPREzy, in a randomized controlled trial using a manikin model. Resuscitation.

[38] Beckers SK, Skorning MH, Fries M, Bickenbach J, Beuerlein S, Derwall M (2007). CPREzy™ improves performance of external chest compressions in simulated cardiac arrest. Resuscitation.

[39] Elding C, Baskett P, Hughes A (1998). The study of the effectiveness of chest compressions using the CPR-plus. Resuscitation.

[40] Peberdy MA, Silver A, Ornato JP (2009). Effect of caregiver gender, age, and feedback prompts on chest compression rate and depth. Resuscitation.

[41] Kämäräinen A, Sainio M, Olkkola KT, Huhtala H, Tenhunen J, Hoppu S (2012). Quality controlled manual chest compressions and cerebral oxygenation during in-hospital cardiac arrest. Resuscitation.

[42] Lukas RP, Sengelhoff C, Döpker S, Harding U, Mertens P, Osada N (2010). Chest compression quality: can feedback technology help? [in German]. Anaesthesist.

[43] Perkins GD, Kocierz L, Smith SC, McCulloch RA, Davies RP (2009). Compression feedback devices over estimate chest compression depth when performed on a bed. Resuscitation.

[44] Hellevuo H, Sainio M, Huhtala H, Olkkola KT, Tenhunen J, Hoppu S (2014). The quality of manual chest compressions during transport – effect of the mattress assessed by dual accelerometers. Acta Anaesthesiol Scand.

[45] Beesems SG, Koster RW (2014). Accurate feedback of chest compression depth on a manikin on a soft surface with correction for total body displacement. Resuscitation.

[46] Perkins GD, Augre C, Rogers H, Allan M, Thickett DR (2005). CPREzy™: an evaluation during simulated cardiac arrest on a hospital bed. Resuscitation.

[47] van Berkom PF, Noordergraaf GJ, Scheffer GJ, Noordergraaf A (2008). Does use of the CPREzy™ involve more work than CPR without feedback?. Resuscitation.

[48] Ameryoun A, Meskarpour-Amiri M, Dezfuli-Nejad ML, Khoddami-Vishteh H, Tofighi S (2011). The assessment of inequality on geographical distribution of non-cardiac intensive care beds in Iran. Iran J Public Health.

[49] Meskarpour-Amiri M, Mehdizadeh P, Barouni M, Dopeykar N, Ramezanian M (2014). Assessment the trend of inequality in the distribution of intensive care beds in Iran: using GINI index. Glob J Health Sci.

[50] Wallace DJ, Angus DC, Seymour CW, Barnato AE, Kahn JM (2015). Critical care bed growth in the United States: a comparison of regional and national trends. Am J Respir Crit Care Med.

